# On three species of the spider genus *Pimoa* (Araneae, Pimoidae) from China

**DOI:** 10.3897/zookeys.855.33501

**Published:** 2019-06-13

**Authors:** Xiaoqing Zhang, Shuqiang Li

**Affiliations:** 1 Institute of Zoology, Chinese Academy of Sciences, Beijing 100101, China Institute of Zoology, Chinese Academy of Sciences Beijing China

**Keywords:** Asia, description, diagnosis, taxonomy

## Abstract

Two new species of the spider genus *Pimoa* Chamberlin & Ivie, 1943 are described from Hunan and Yunnan Provinces, China: *P.binchuanensis***sp. nov.** (♂♀) and *P.xinjianensis***sp. nov.** (♂♀). In addition, the male of *P.lata* Xu & Li, 2009 is described for the first time. The DNA barcodes of the two new species are documented.

## Introduction

Pimoidae Wunderlich, 1986 is a relatively small family, with 44 described species belonging to four genera (Li and Quan 2017; WSC 2019). *Pimoa* Chamberlin & Ivie, 1943 is the largest genus of the family, with 33 valid species. As a relict group, it has disjunct range and occurs in the western Nearctic (from Washington to California, USA), the western Mediterranean and Asia (from Himalaya to Beijing) (Li and Lin 2016; WSC 2019). Fifteen *Pimoa* species are known from Asia so far, nine from China, four from India and two from Nepal. Most of these species are well described in revisions (Hormiga 1994a; Xu and Li 2007). While studying material from Southwest China, we found two new species and the unknown male of *P.lata* Xu & Li, 2009. The goal of this paper is to provide descriptions of the new species and the unknown male.

## Material and methods

Specimens were examined with a LEICA M205C stereomicroscope. Images were captured with an Olympus C7070 wide zoom digital camera (7.1 megapixels) mounted on an Olympus SZX12 dissecting microscope. Epigynes and male palps were examined after dissection from the spiders’ bodies. The left palps were illustrated unless otherwise noted. Epigynes were removed and treated in a warmed 10% potassium hydroxide (KOH) solution.

All measurements were obtained using a LEICA M205C stereomicroscope and are given in millimeters. Eye sizes are measured as the maximum diameter from either dorsal or frontal views. Leg measurements are shown as: Total length (femur, patella + tibia, metatarsus, tarsus). The terminology used in the text and the figure legends follows Hormiga (1994a). Distribution maps were generated using ArcView GIS (ESRI) software.

Abbreviations used in this paper and in the figure legends: **ALE** = anterior lateral eye; **AME** = anterior median eye; **AME-ALE** = distance between **AME** and **ALE**; **AME-AME** = distance between **AME** and **AME**; **ALE-PLE** = distance between **ALE** and **PLE**; **AS** = alveolar sclerite; **C** = conductor; **CO** = copulatory opening; **CP** = cymbial process; **CS** = cymbial sclerite; **DP** = dorsal plate of the epigyne; **E** = embolus; **EP** = embolic process; **ET** = embolic tooth; **FD** = fertilization duct; **MA** = median apophysis; **P** = paracymbium; **PLE** = posterior lateral eye; **PME** = posterior median eye; **PME-PLE** = distance between **PME** and **PLE**; **PME-PME** = distance between **PME** and **PME**; **S** = spermatheca; **T** = tegulum; **VP** = ventral plate of epigyne.

DNA barcodes were obtained for the two new species by amplifying and sequencing a partial fragment of the mitochondrial gene cytochrome oxidase subunit I (COI) using primers LCO1490-oono (5’-CWACAAAYCATARRGATATTGG-3’) (Folmer et al. 1994; Miller et al. 2010) and HCO2198-zz (5’-TAAACTTCCAGGTGACCAAAAAATCA-3’) (Folmer et al. 1994; Zhao and Li 2017). For additional information on extraction, amplification and sequencing procedures, see Wang et al. (2008). All sequences were checked using BLAST and are deposited in GenBank. The accession numbers are provided in Table 1.

**Table 1.** Voucher specimen information.

**Species**	**GenBank accession number**	**Sequence length**	**Collection localities**
*Pimoabinchuanensis* sp. nov.	MK910743	609bp	Binchuan County, Yunnan, China
*Pimoaxinjianensis* sp. nov.	MK910744	609bp	Longshan County, Hunan, China

All specimens (including molecular vouchers) are deposited in the Institute of Zoology, Chinese Academy of Sciences (**IZCAS**), Beijing, China.

## Taxonomy

### Family Pimoidae Wunderlich, 1986

#### Genus Pimoa

Taxon classificationAnimaliaAraneaePimoidae

Chamberlin & Ivie, 1943

Pimoa : Chamberlin and Ivie 1943: 9; Hormiga 1994a: 4; Hormiga and Lew 2014: 1; Mammola et al. 2016: 1.

##### Type species.

*Labullahespera* Gertsch & Ivie, 1936, from California, USA.

##### Diagnosis.

**Figure 1.**
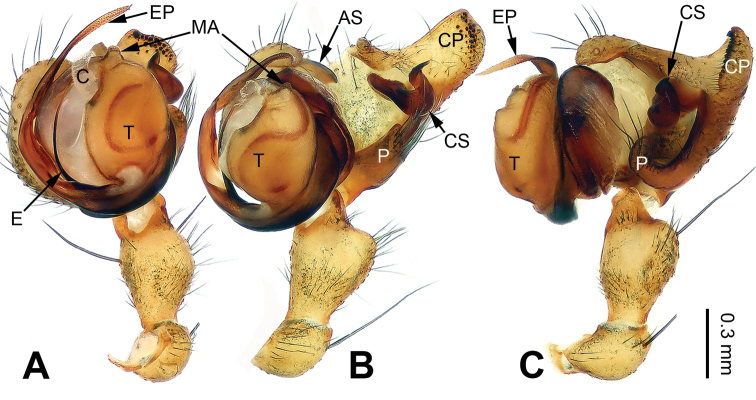
Left palp of *Pimoabinchuanensis* sp. nov., holotype **A** prolateral view **B** ventral view **C** retrolateral view. Abbreviations: **AS** = alveolar sclerite; **C** = conductor; **CP** = cymbial process; **CS** = cymbial sclerite; **E** = embolus; **EP** = embolic process; **MA** = median apophysis; **P** = paracymbium; **T** = tegulum. Scale bar: equal for **A, B** and **C**.

**Figure 2.**
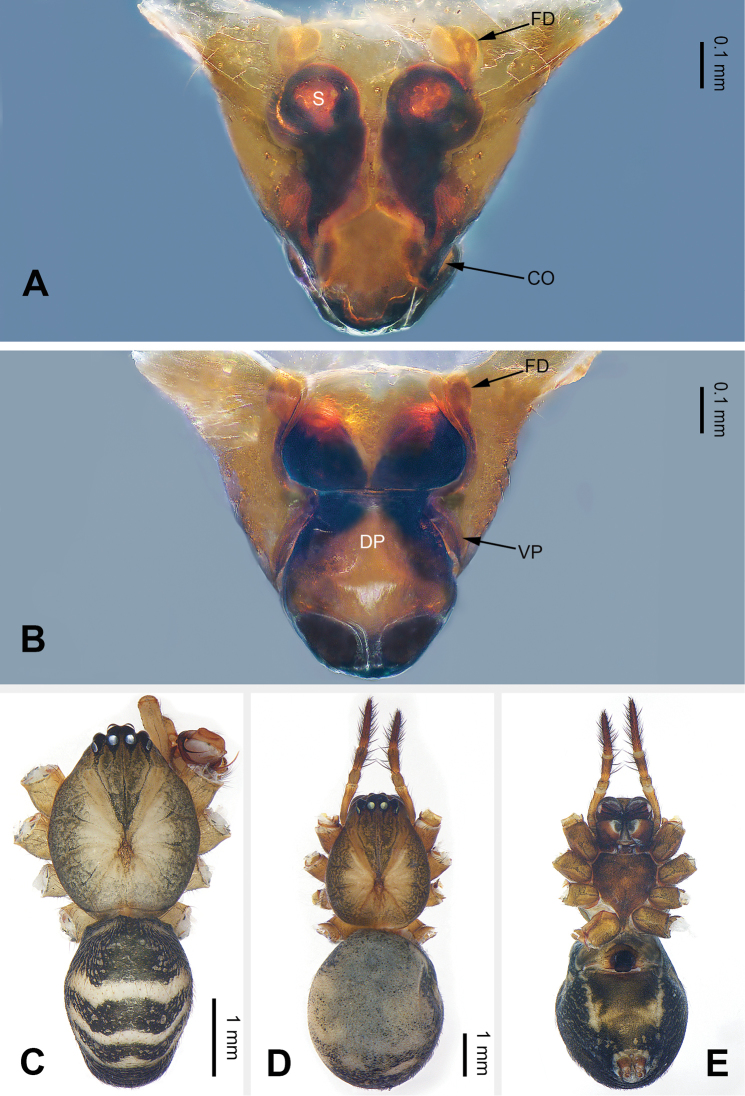
Epigyne and habitus of *Pimoabinchuanensis* sp. nov., female paratype and male holotype **A** epigyne, ventral view **B** vulva, dorsal view **C** male habitus, dorsal view **D** female habitus, dorsal view **E** female habitus, ventral view. Abbreviations: **CO** = copulatory opening; **DP** = dorsal plate of the epigyne; **FD** = fertilization duct; **S** = spermatheca; **VP** = ventral plate of epigyne. Scale bars: equal for **D** and **E**.

Males of *Pimoa* can be distinguished from *Weintrauboa* Hormiga, 2003 by the elongate cymbial process (CP) with many cuspules (vs cymbial process (CP) and cuspules absent) (Fig. 1A–C; Hormiga 2003: figs 1, 2). From *Putaoa* Hormiga & Tu, 2008, it can be distinguished by the absence of distinctly large macrosetae on the palpal tibia (vs presence of a large macroseta) (Fig. 1A–C; Hormiga and Tu 2008: figs 3, 5–6). Females of *Pimoa* can be distinguished from *Weintrauboa* by the protruding epigyne with a distinct dorsal plate (DP) (vs dorsal plate absent) (Fig. 2A, B; Hormiga 2003: figs 2–3). From *Putaoa*, it can be distinguished by the absence of lateral openings on the epigyne (vs two distinct lateral openings) (Fig. 2A, B; Hormiga and Tu 2008: figs 2, 4, 8).

##### Composition.

Thirty-three valid species of *Pimoa* are currently known from the western Nearctic (14), western Mediterranean (4) and South Asia (15) (WSC 2019).

#### Pimoa binchuanensis sp. nov.

Taxon classificationAnimaliaAraneaePimoidae

http://zoobank.org/9DC874E9-8DC9-4782-BAF5-4E9397878C0C

Figs 1 , 2 , 7

##### Type material.

**Holotype** ♂ (IZCAS-Ar39293): China: Yunnan: Dali Bai Autonomous Prefecture: Binchuan County: Jizushan Town, Mt. Jizu, 25.9667°N, 100.3746°E, 2568±4 m, 25.III.2019, Z. Chen. **Paratype**: ♀ (IZCAS-Ar39294): same area, 25.9639°N, 100.3712°E, 2658 m, 1.XII.2014, Y. Li & Z. Chen.

##### Etymology.

The specific name refers to the type locality; adjective.

##### Diagnosis.

The male of *P.binchuanensis* sp. nov. can be easily distinguished from other congeners, except for *P.anatolica* Hormiga, 1994 and *P.lihengae* Griswold, Long & Hormiga, 1999, by having a long and complex cymbial sclerite (CS) and an elongate cymbial process (CP). From *P.anatolica* and *P.lihengae*, it can be distinguished by the long embolic process (EP), about 2 times longer than the embolus (vs embolic process shorter than embolus) (cf. Fig. 1A–C; Griswold et al. 1999: figs 15–17; Xu and Li 2007: figs 4–8). The female of *P.binchuanensis* sp. nov. can be distinguished from other congeners by having a broad dorsal plate (DP) of the epigyne with an oval tip and trapezoidal basal part (vs dorsal plate narrow or indistinct) (Fig. 2A, B).

##### Description.

**Male** (holotype, IZCAS-Ar39293): Total length 5.25. Carapace 2.50 long, 2.00 wide. Abdomen 2.75 long, 1.75 wide. Eye sizes and interdistances: AME 0.20, ALE 0.16, PME 0.15, PLE 0.20; AME-AME 0.05, AME-ALE 0.05, PME-PME 0.10, PME-PLE 0.10. Leg measurements: I: missing; II: 17.00 (5.00, 5.25, 5.00, 1.75); III: 10.75 (3.25, 3.50, 3.00, 1.00); IV: missing. Promargin of chelicerae with 2 teeth, retromargin with 1 tooth. Carapace yellowish, with black lateral margins, the thoracic fovea and radial grooves distinct, sternum yellowish, nearly almond-shaped. Abdomen brownish with yellow transverse bands, nearly oval. Legs yellowish with black annulations. Palp: patella short, about 1/2 of tibial length; tibia long, about 1/2 of cymbial length; paracymbium short, about 1/3 of cymbial length, somewhat hook-shaped; cymbial sclerite (CS) long, about 1/2 of cymbial length, spindle-shaped; cymbial process (CP) broad and long, about 1/2 of cymbial length, with more than 20 cuspules; median apophysis (MA) indistinct; conductor distinct; embolic process (EP) long, about 1.5 times as long as embolus, tip with fine granulations; embolus bent and long, about the same length as the cymbium, beginning at the 7:30 o’clock position; embolic tooth absent (Fig. 1A–C).

**Female**: (paratype, IZCAS-Ar39294): Total length 7.12. Carapace 3.16 long, 2.47 wide. Abdomen 3.96 long, 3.28 wide. Eye sizes and interdistances: AME 0.15, ALE 0.20, PME 0.17, PLE 0.17; AME-AME 0.14, AME-ALE 0.11, PME-PME 0.14, PME-PLE 0.22. Leg measurements: I: 19.78 (5.71, 6.86, 5.06, 2.15); II: 16.23 (4.94, 5.26, 4.23, 1.80); III: 11.47 (3.52, 3.56, 3.08, 1.31); IV: 15.64 (4.87, 5.13, 4.10, 1.54). Promargin and retromargin of chelicerae with 3 teeth. Carapace brownish, the thoracic fovea and radial grooves distinct, sternum yellowish, and shield-shaped. Abdomen greyish, somewhat oval, transverse bands indistinct. Legs brownish without annulations. Epigyne: triangular; ventral (VP) and dorsal plates (DP) broad, length subequal to width; copulatory openings hidden; spermathecae globose, separated by about half of the radius; fertilization ducts laterally oriented (Fig. 2).

##### Distribution.

Type locality only, Yunnan, China (Fig. 7).

#### Pimoa lata

Taxon classificationAnimaliaAraneaePimoidae

Xu & Li, 2009

Figs 3 , 4 , 7

Pimoa lata Xu & Li, 2009: 56, figs 1–8 (♀).

##### Type material.

**Holotype** ♀: China: Sichuan: Lushan County: Weita Village, Shuiluodong Cave, (30.28°N, 102.97°E, 1338 m), 15.X.2005, S. Li.

##### Other material examined.

2♀1♂ (IZCAS-Ar39295-Ar39297): China: Sichuan: Lushan County: Weita Village, Shuiluodong Cave, 30.2750°N, 102.9690°E, 1302 m, 27.VI.2018, X. Zhang.

##### Diagnosis.

**Figure 3.**
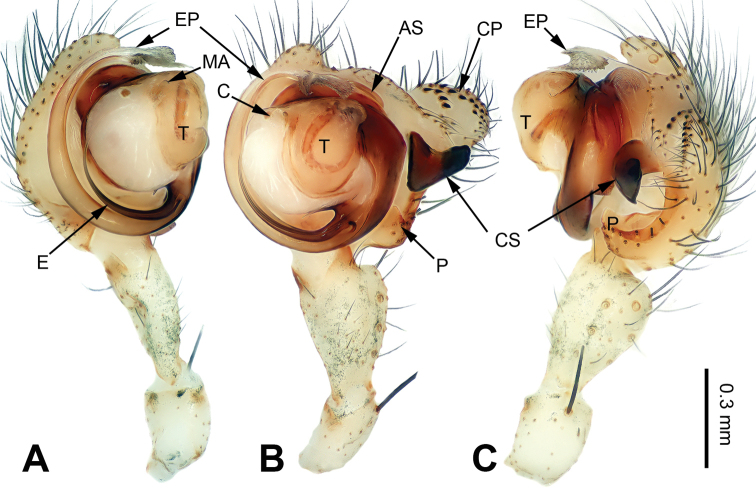
Left palp of *Pimoalata***A** prolateral view **B** ventral view **C** retrolateral view Scale bar: Abbreviations: **AS** = alveolar sclerite; **C** = conductor; **CP** = cymbial process; **CS** = cymbial sclerite; **E** = embolus; **EP** = embolic process; **MA** = median apophysis; **P** = paracymbium; **T** = tegulum. Scale bar: equal for **A, B** and **C**.

**Figure 4.**
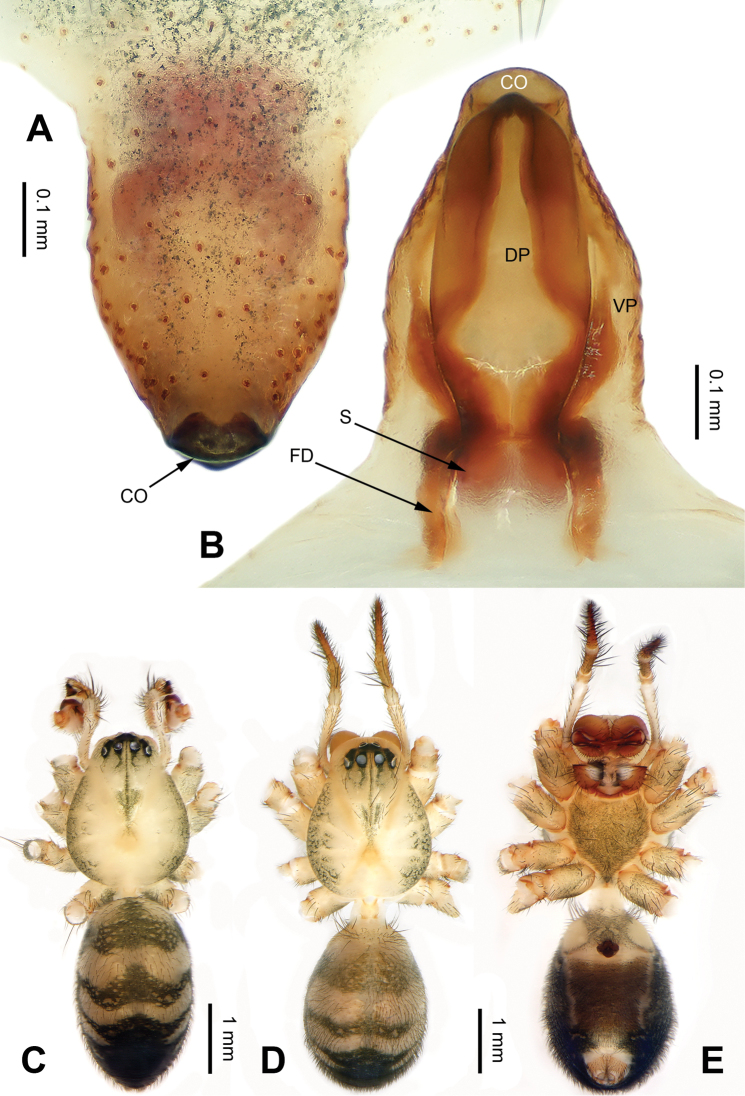
Epigyne and habitus of *Pimoalata***A** epigyne, ventral view **B** vulva, dorsal view **C** male habitus, dorsal view **D** female habitus, dorsal view **E** female habitus, ventral view. Abbreviations: **CO** = copulatory opening; **DP** = dorsal plate of the epigyne; **FD** = fertilization duct; **S** = spermatheca; **VP** = ventral plate of epigyne. Scale bars: equal for **D** and **E**.

The male of *P.lata* can be easily distinguished from other congeners, except for *P.reniformis* Xu & Li, 2007 and *P.trifurcata* Xu & Li, 2007 by having a short paracymbium and a large and blunt cymbial process (CP), with many cuspules. From *P.reniformis*, it can be distinguished by the short cymbial sclerite (CS), about 1/3 of the cymbial length, with a blunt tip (vs a long cymbial sclerite (CS) in *P.reniformis*, about 1/2 of cymbial length, with a sharp tip). From *P.trifurcata*, it can be distinguished by the bifurcated embolic process (EP) (vs the embolic process (EP) in *P.trifurcata* with a trifurcate tip) (cf. Fig. 3A–C; Xu and Li 2007: figs 38–41, 49–54). The female of *P.lata* can be distinguished from all other congeners by the lip-shaped dorsal plate (DP) (vs dorsal plate narrow or indistinct) (Fig. 4A, B).

##### Description.

**Male** (IZCAS-Ar39295): Total length 5.00. Carapace 2.25 long, 2.00 wide. Abdomen 2.75 long, 1.75 wide. Eye sizes and interdistances: AME 0.15, ALE 0.16, PME 0.15, PLE 0.16; AME-AME 0.05, AME-ALE 0.05, PME-PME 0.10, PME-PLE 0.10. Leg measurements: I: 21.00 (6.00, 6.75, 5.50, 2.75); II: 18.75 (5.25, 6.50, 5.00, 2.00); III: 13.40 (4.25, 4.00, 3.50, 1.65); IV: 15.00 (5.00, 4.75, 4.00, 1.25). Promargin and retromargin of chelicerae with 3 teeth. Carapace yellowish, with green lateral margins, the thoracic fovea distinct, sternum yellowish. Abdomen brownish with yellow transverse bands. Legs yellowish, with black annulations. Palp: patella short, about 1/3 of tibial length; tibia long, almost as long as cymbial length; paracymbium short, about 1/5 of cymbial length, with a blunt tip; cymbial sclerite (CS) short, about 1/3 of cymbial length, with a blunt, black tip; cymbial process (CP) broad and short, about 1/3 of cymbial length, with more than 15 cuspules; median apophysis (MA) indistinct; conductor indistinct; embolic process (EP) long, about 2 times as long as embolus, with two jagged tips; embolus bent and long, about the same length as the cymbium, beginning at 5:30 o’clock position; embolic tooth indistinct (Fig. 3A–C).

**Female** (Fig. 4). Description see Xu and Li (2009: figs 1–8).

##### Distribution.

Shuiluodong Cave, Sichuan, China (Fig. 7).

##### Remark.

The male of this species is described for the first time.

#### Pimoa xinjianensis sp. nov.

Taxon classificationAnimaliaAraneaePimoidae

http://zoobank.org/C804AFD1-875B-4960-8EBC-DC77C30929D7

Figs 5 , 6 , 7

##### Type material.

**Holotype** ♂ (IZCAS-Ar39298): China: Hunan: Xiangxi Tujia and Miao Autonomous Prefecture: Longshan County: Xichehe Town: Xinjian Village, Xianren Cave, 29.0855°N, 109.5109°E, 503 m, 26.X.2018, X. Zhang & Z. Chen. **Paratypes**: 3♀ (IZCAS-Ar39299-Ar39301), same data as holotype; 2♀1♂ (IZCAS-Ar39302-Ar39304), Longshan County: Xichehe Town: Shuitong Village, Yangjia Cave, 29.0879°N, 109.4945°E, 431 m, 26.X.2018, X. Zhang & Z. Chen; 2♀ (IZCAS-Ar39305-Ar39306), Longshan County: Wuya Town: Xiyan Village, Mt. Tianma, unnamed cave, 29.5701°N, 109.7051°E, 840 m, 28.X.2018, X. Zhang & Z. Chen.

##### Etymology.

The specific name refers to the type locality; adjective.

##### Diagnosis.

**Figure 5.**
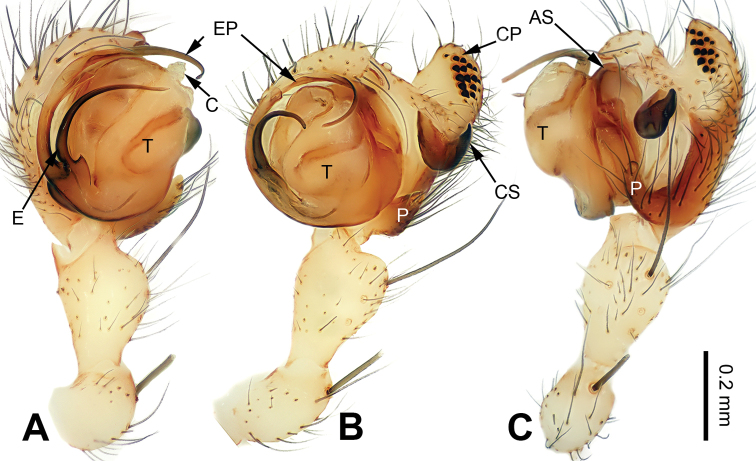
Left palp of *Pimoaxinjianensis* sp. nov., holotype **A** prolateral view **B** ventral view **C** retrolateral view. Abbreviations: **AS** = alveolar sclerite; **C** = conductor; **CP** = cymbial process; **CS** = cymbial sclerite; **E** = embolus; **EP** = embolic process; **P** = paracymbium; **T** = tegulum. Scale bar: Equal for **A, B** and **C**.

**Figure 6.**
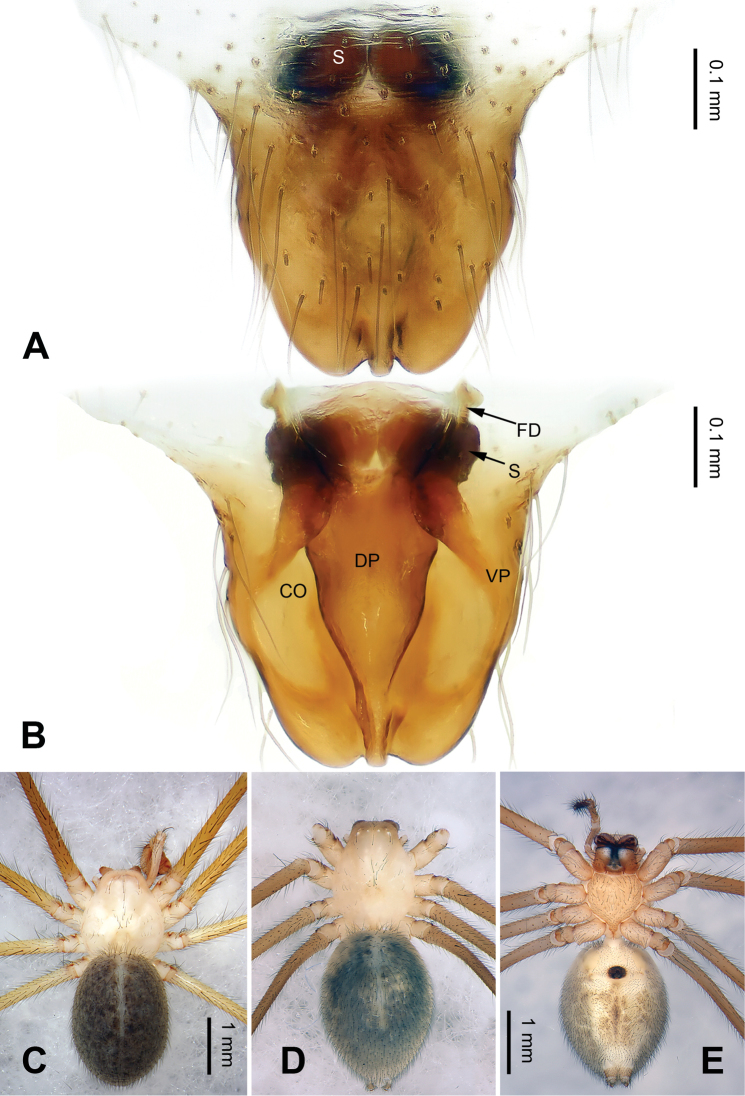
Epigyne and habitus of *Pimoaxinjianensis* sp. nov., female paratype and male holotype **A** epigyne, ventral view **B** vulva, dorsal view **C** male habitus, dorsal view **D** female habitus, dorsal view **E** female habitus, ventral view. Abbreviations: **CO** = copulatory opening; **DP** = dorsal plate of the epigyne; **FD** = fertilization duct; **S** = spermatheca; **VP** = ventral plate of epigyne. Scale bars: Equal for **D** and **E**.

Both sexes of *P.xinjianensis* sp. nov. can be easily distinguished from other congeners by the strongly reduced (vestigial) eyes. The palp of *P.xinjianensis* sp. nov. can be distinguished from that of other congeners by the long embolic process (EP), about 2 times longer than the embolus (vs a short embolic process, almost as long as the embolus) (cf. Figs 5, 6). The epigyne of *P.xinjianensis* sp. nov. differs from other congeners by having distinct (unhidden) copulatory openings (vs hidden or indistinct) (Fig. 6).

##### Description.

**Male** (holotype, IZCAS-Ar39298): Total length 3.75. Carapace 1.50 long, 1.30 wide. Abdomen 2.25 long, 1.70 wide. Eyes vestigial (strongly reduced in size). Leg measurements: I: 21.50 (6.50, 6.00, 6.75, 2.25); II: 20.20 (6.00, 5.75, 6.15, 2.30); III: 17.40 (4.75, 5.50, 5.15, 2.00); IV: 20.00 (6.00, 6.75, 5.50, 1.75). Promargin of chelicerae with 3 teeth, retromargin with 2 teeth. Carapace yellowish, the thoracic fovea distinct, sternum yellowish. Abdomen brownish. Legs yellowish, without annulations. Palp: patella short, about 1/2 of tibial length; tibia long, almost as long as cymbial length; paracymbium short, about 1/3 of cymbial length, with rounded tip; cymbial sclerite (CS) short, about 1/3 of cymbial length, with a tapering, black tip; cymbial process (CP) long, about 1/2 of the cymbial length, with more than 13 cuspules; median apophysis (MA) indistinct; embolic process (EP) long, about 2 times longer than the embolus, tip without granulation; embolus bent and long, about 1/2 of cymbial length, beginning at 6:30 o’clock position; embolic tooth distinct (Fig. 5A–C).

**Female** (paratype, IZCAS-Ar39299): Total length 4.75. Carapace 1.75 long, 1.50 wide. Abdomen 3.00 long, 2.25 wide. Eyes vestigial (only with 6 white spots). Leg measurements: I: 20.60 (6.20, 6.00, 6.10, 2.30); II: 19.30 (5.95, 5.75, 5.50, 2.10); III: 14.75 (4.75, 4.50, 4.00, 1.50); IV: 18.35 (6.10, 5.25, 5.00, 2.00). Cheliceral teeth as in male. Carapace yellowish; sternum flavescent. Abdomen greyish. Legs reddish, without annulations. Epigyne: trapezoidal; ventral plate (VP) broad, length subequal to width; dorsal plate (DP) triangular; copulatory openings distinct; spermathecae oval, touching each other; fertilization ducts medially oriented (Fig. 6).

##### Distribution.

**Figure 7.**
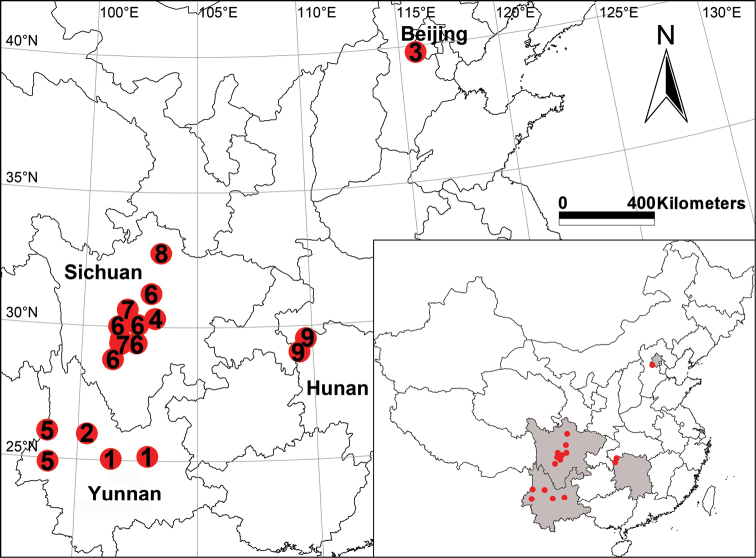
Distribution records of Pimoa species from China **1***P.anatolica***2***P.binchuanensis* sp. nov. **3***P.clavata***4***P.lata***5***P.lihengae***6***P.reniformis***7***P.trifurcata***8***P.wanglangensis***9***P.xinjianensis* sp. nov.

Type locality only, Hunan, China (Fig. 7).

## Supplementary Material

XML Treatment for Pimoa

XML Treatment for Pimoa binchuanensis

XML Treatment for Pimoa lata

XML Treatment for Pimoa xinjianensis
